# Protein-Protein Interaction Antagonists as Novel Inhibitors of Non-Canonical Polyubiquitylation

**DOI:** 10.1371/journal.pone.0011403

**Published:** 2010-06-30

**Authors:** Johanna Scheper, Marta Guerra-Rebollo, Glòria Sanclimens, Alejandra Moure, Isabel Masip, Domingo González-Ruiz, Nuria Rubio, Bernat Crosas, Óscar Meca-Cortés, Noureddine Loukili, Vanessa Plans, Antonio Morreale, Jerónimo Blanco, Angel R. Ortiz, Àngel Messeguer, Timothy M. Thomson

**Affiliations:** 1 Department of Cell Biology, Institute for Molecular Biology (IBMB-CSIC), Barcelona, Spain; 2 Department of Biological Organic Chemistry, Institute for Advanced Chemistry of Catalonia (IQAC-CSIC), Barcelona, Spain; 3 Bioinformatics Unit, Centro de Biología Molecular Severo Ochoa (CBM-UAM-CSIC), Madrid, Spain; 4 Catalan Center for Cardiovascular Research (CIC-CSIC), and CIBER de Bioingeniería, Biomateriales y Nanomedicina, Barcelona, Spain; Universidade Federal do Rio de Janeiro (UFRJ), Brazil

## Abstract

**Background:**

Several pathways that control cell survival under stress, namely RNF8-dependent DNA damage recognition and repair, PCNA-dependent DNA damage tolerance and activation of NF-κB by extrinsic signals, are regulated by the tagging of key proteins with lysine 63-based polyubiquitylated chains, catalyzed by the conserved ubiquitin conjugating heterodimeric enzyme Ubc13-Uev.

**Methodology/Principal Findings:**

By applying a selection based on *in vivo* protein-protein interaction assays of compounds from a combinatorial chemical library followed by virtual screening, we have developed small molecules that efficiently antagonize the Ubc13-Uev1 protein-protein interaction, inhibiting the enzymatic activity of the heterodimer. In mammalian cells, they inhibit lysine 63-type polyubiquitylation of PCNA, inhibit activation of NF-κB by TNF-α and sensitize tumor cells to chemotherapeutic agents. One of these compounds significantly inhibited invasiveness, clonogenicity and tumor growth of prostate cancer cells.

**Conclusions/Significance:**

This is the first development of pharmacological inhibitors of non-canonical polyubiquitylation that show that these compounds produce selective biological effects with potential therapeutic applications.

## Introduction

Modifications by ubiquitin (ubiquitylation) control the fate and participation of proteins in fundamental biological processes [1]. The ubiquitylation of a protein involves the formation of a isopeptide bond between a substrate lysine residue and the carboxy terminal Gly76 on ubiquitin. Ubiquitin is activated by an ATP-hydrolyzing ubiquitin-activating enzyme (Uba or E1), that forms a high energy thioester bond between a Cys of its active site and the carboxy terminus of ubiquitin. Activated ubiquitin is transferred to a ubiquitin-conjugating enzyme (Ubc or E2) and a thioester-linked E2-ubiquitin complex is formed. Finally, E2 interacts with a ubiquitin-protein ligase (E3), which conjugates ubiquitin to the substrate protein and confers substrate specificity to the pathway. Ubiquitin has several lysine residues that may be substrates themselves of ubiquitylation, leading to the formation of polyubiquitin chains. The signaling properties of ubiquitylation vary according to the topology of polyubiquitin chains, which depends on the particular lysine residue on the ubiquitin molecule used to form these chains [2]. Thus, polyubiquitin chains linked through K48 (often dubbed as “canonical”) are recognized by specific subunits of the 26S proteasome regulatory particle, leading to the degradation of the modified protein [1], [2]. Polyubiquitin chains based on K63 are not as efficiently recognized by the proteasome, and rather modify substrate proteins for interactions with other proteins that participate in signaling and other nonproteolytic processes [2], [3]. The formation of this class of “non-canonical” polyubiquitin chains is mostly catalyzed by the heterodimeric ubiquitin conjugating enzyme formed by Ubc13 and a Uev protein, Uev1 or Uev2/Mms2 in higher eukaryotes, or Mms2 in the yeast S. cerevisiae [2], [4], [5]. The N-terminal alpha helix of Uev1 (or Mms2) engages in high affinity interactions with a hydrophobic groove on Ubc13 [6], [7], [8], [9]. A critical contributor to the affinity and specificity of this interaction is Phe13 in Uev1, which fits into a deep pocket formed by residues Glu55, Leu56, Phe57 and Arg70 of Ubc13 [6], [7], [8]. Although other residues contribute to heterodimerization, the above configuration accounts for most of the specificity and affinity of the interaction between Uev1 and Ubc13 [8], [9], [10].

In the yeast *S. cerevisiae*, DNA damage induces K63 polyubiquitylation of the polymerase auxiliary factor PCNA, promoting its function in the error-free DNA damage response pathway, a process dependent on Ubc13 and Mms2 [11], [12], [13], which is conserved in mammalian cells [14],[15]. Of the two Uev proteins in mammals, Uev2/Mms2, but not Uev1, appears to be specifically involved in DNA damage repair [16]. The Ubc13-Uev2 heterodimer, recruited by the ubiquitin ligases RNF8 and RNF168, also promotes the recruitment of the BRCA1 A DNA damage repair complex, and K63 polyubiquitylation of histones H2A and H2AX are critical in this process [12]. One of the best studied processes regulated by K63 polyubiquitylation in mammals are signaling pathways that activate the transcriptional factor NF-κB [17]. Upon binding of TNF-α to its receptor, the RING finger E3 ubiquitin ligase cIAP is recruited to the receptor complex and ubiquitylates RIP1 possibly through Ubc13-dependent K63 polyubiquitination, resulting in the recruitment of LUBAC, and the complexes TAK1/TAB2/TAB2 and NEMO/IKKα/IKKβ [18]. LUBAC drives linear polyubiquitylation of several components of the TNF-R1 complex [19], which promotes the stabilization of the complex [20] and is essential for the recruitment of NEMO and activation of NF-κB [21]. Binding of IL-1β to IL-1R recruits TRAF6 which oligomerizes, self-polyubiquitylates in a reaction catalyzed by Ubc13-Uev1 and recruits the TAK1/TAB2/TAB2 and NEMO/IKKα/IKKβ complexes. Both cytokines eventually activate a kinase cascade that leads to the phosphorylation-dependent ubiquitylation and degradation of IκB, allowing the nuclear translocation and activation of NF-κB [17]. Recent evidence indicates that, in addition to Ubc13, UbcH5 can also mediate K63 polyubiquitylation of RIP, and that NF-κB activation by TNF-α may not be as dependent on K63 polyubiquitylation as previously thought [22]. K63 polyubiquitylation is also critical for the activation of additional signaling pathways [23], [24], and has been shown to regulate receptor endocytosis and processing [25], [26], protein sorting in the multivesicular body pathway [27], cell motility [28], cell-cycle checkpoints [29], and autophagy [30].

Therefore, Ubc13-dependent K63 polyubiquitylation critically regulates processes that generally enhance the survival of cells and organisms in response to certain forms of stress, such as DNA damage or exposure to infectious agents. A pharmacological inhibitor of this modification would be useful not only to study the proteins and biochemical and cellular processes that are modified by this signal, but also to modulate these pathways for therapeutic purposes: for instance, to sensitize cancer cells to DNA damaging agents, or to blunt excessive immune activation.

## Results

### Development of antagonists of the Ubc13-Uev1 interaction

**Figure 1:**
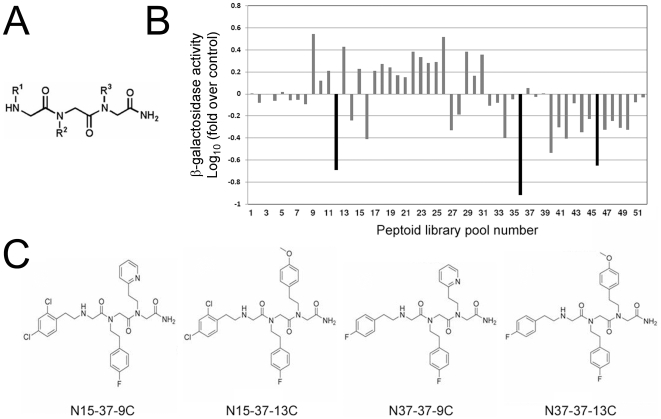
Peptoid combinatorial libraries as sources of structures that antagonize protein-protein interactions. (**A**) General structure of the combinatorial *N*-alkyl glycine library. (**B**) Yeast two-hybrid screening for peptoid pools that inhibit the Ubc13-Uev1 interaction. As a control interaction, the p53-large T interaction was assayed with the same pools in parallel assays. The β-galactosidase activity was normalized to the values for the control interaction, and to the values for the Ubc13-Uev1 interaction in the presence of solvent only (without peptoids). (**C**) Structures of the individual peptoids inferred as the most likely active inhibitors of the Ubc13-Uev1 interaction.

The hydrophobic grooves and pocket on Ubc13 that serve as its interface with Uev1 present features of a candidate site for specific and high-affinity occupancy by small molecules [31]: a relatively small and deep surface, well-delimited by residues that are not highly polar on the average. Preliminary docking analyses using a comprehensive peptide-based structural library had suggested us that this pocket could be effectively occupied by peptides with the preferred consensus sequence NH_3_-X – Pro – β-strand aa (Val/Leu/Ile/Arg/Cys) – hydrophobic/aromatic – X – Pro-COOH (M. Murcia & A.R.O., unpublished observations). We reasoned that peptidomimetic molecules capable of occupying this pocket with sufficient affinity could compete with Uev1 for its interaction with Ubc13, thereby inhibiting the enzymatic activity of the heterodimer. We used a combinatorial chemical library based on trimers of N-alkylglycines (peptoids) [32], [33] as an initial source of peptidomimetic structures. Peptoids are characterized by a peptide scaffold with side chains attached to the backbone nitrogen atoms [32], which confers them with several structural properties of peptides [34], [35], together with the desirable pharmacological property of being more resistant to proteolytic enzymes [36]. The combinatorial arrangement of N-substituted free amines provided the necessary chemical diversity, and the positional scanning format adopted for this particular library afforded a convenient screening scheme with 52 pools, each containing 320 (pools 1–20) or 256 individual peptoids (pools 21–52), for a total of 5,120 compounds [33]. Conformational flexibility greatly multiplies the structural diversity provided by this library [37], [38], a feature that we deliberately chose in spite of the predicted detriment to high-affinity interactions with specific targets of such flexible structures. The general structure of the peptoid library used in this screening is shown in Fig. 1A.

As a system to test the capacity of peptoids to inhibit the interaction of Ubc13 with Uev1, we used the yeast-two hybrid assay, since it permits to readily determine the specificity of the inhibitory activities on a fully defined protein-protein interaction and, simultaneously, it constitutes a stringent filter for the bioavailability of active molecules. Yeast cells bearing interacting human Ubc13 and Uev1 were used to screen the combinatorial peptoid library pools for consistent and significant inhibition of this interaction. As a control, an unrelated protein-protein interaction was tested, that of p53 with SV40 large T. Of the 52 pools, those numbered 12, 36 and 46 produced the greatest inhibitory activity on the Ubc13-Uev1 interaction, without significant inhibition on the control p53-large T interaction (Fig. 1B). Deconvolution of the selected pools (i.e., 12 and 16 from the sublibrary where R1 was known, 36 from the sublibrary where R2 was known, and 42 and 46 from the sublibrary where R3 was known) indicated that the inhibitory activity corresponded to four preferred structures (Fig. 1C). The selected amines in these peptoids are: at position R1, either 4′-fluorophenylethyl (peptoid N37-37-9C) or 2′-4′-dichlorophenylethyl (N15-37-13C); at position R2, 4′-fluorophenylethyl; at position R3, either 4′-methoxyphenylethyl (N37-37-13C and N15-37-13C) or 2-(2′-pyridyl)ethyl (N37-37-9C and N15-37-9C).

**Figure 2:**
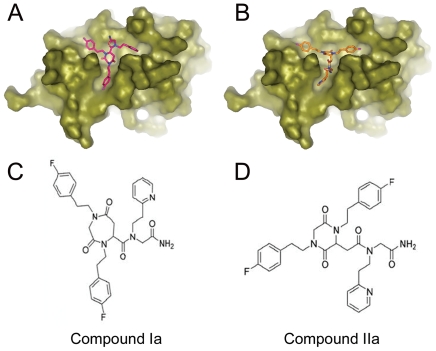
Selection of cyclic structures with optimal fitting on Ubc13 by large-scale molecular docking. (**A**, **B**) Connolly surface representation of Ubc13 along with the conformers of cyclic structures with highest ranked energy and fitting values for docking on the surface of Ubc13 (**A**, family I cyclicization mode, enantiomer 2; **B**, family II cyclicization mode, enantiomer 1). **C**, Structure of cyclic compound Ia, synthesized after the structure for family I cyclic molecules, derived from peptoid N37-37-9C. **D**, Structure of cyclic compound IIa, synthesized after the structure for family II cyclic molecules, derived from peptoid N37-37-9C.

Our hypothesis is that these peptoids inhibit the Ubc13-Uev1 interaction by occupying the Ubc13 dimerization interface with Uev1. Therefore, we analyzed whether these molecules fit onto the Ubc13 surface by means of molecular docking. Because of the inherent flexibility of the carbon framework and N-substituted bonds in alkylglycines, the selected peptoids present a high degree of conformational heterogeneity. To reduce this conformational flexibility, we evaluated 8 families of cyclic structures that are relatively more rigid than the planar equivalents of the selected peptoids, with the added feature that they are readily suitable for synthesis following standard chemical procedures. All energetically favourable spatial arrangements for these structures were computed. Of the 8 families of cyclic structures, six had 4 enantiomers and two had 2 enantiomers, and each molecule had between 10 and 14 torsional angles. Thus, the total number of ligands generated was ≈5×10^6^. This small structural library was subjected to virtual screening to select the molecules with the best fit to Ubc13. For each ligand, the energetically most favourable orientations for interaction with the Ubc13 surface were computed and ranked with CDOCK [39] (Tables S1 and S2). The ligands with the most favourable interactions corresponded to conformers of cyclic families I and II, both derived from peptoid N37-37-9C. Figures 2A and 2B show Connolly surface representations of the computed interactions of these two compounds with Ubc13. These two structures differ mainly in their cyclical components, with either seven (family I) or six (family II) atom rings, that occupy the center of the hydrophobic pocket on Ubc13, with the side chains adapting with favorable interaction energies to the three grooves that stem from the pocket (Fig. 2A and B). These models suggest that family I compounds have more favorable interaction energies on Ubc13 than family II compound (Tables S1 and S2).

### *In vitro* activities of Ubc13-Uev1 antagonists

**Figure 3:**
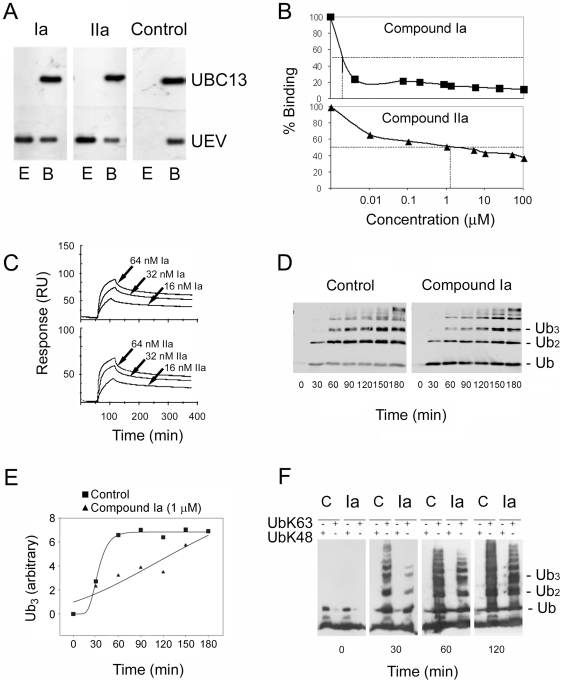
*In vitro* activities of compounds Ia and IIa. (**A**) Inhibition of the Ubc13-Uev1 interaction by compounds Ia and IIa, determined by GST-pull-down assays. Matrix-bound GST-Ubc13, preincubated with the test or control compounds at a final concentration of 100 nM, were allowed to interact with recombinant Uev1. Fraction E corresponds to the eluted fraction of Uev1. Fraction B corresponds to the Uev1 that remaind bound to GST-Ubc13, and that are eluted with reduced glutathione. All fractions were assayed by Western blotting with anti-Ubc13 and anti-Uev antibodies. (**B**) Surface plasmon resonance Ubc13-Uev1 binding competition assay. Ubc13 was pre-incubated with varying concentrations of compounds Ia or IIa and injected into a CM5 chip on which Uev1 had been immobilized. The IC50 for binding inhibition was 10 pM for compound Ia, and 1.1 µM for compound IIa. (**C**) Surface plasmon resonance sensorgrams for the interaction of UBC13 with immobilized compound Ia (top) or IIa (bottom). Lysine derivatives of either compound were immobilized onto CM chips, and Ubc13 was run in the mobile phase at the indicated concentrations. From the signals, dissociation constants for Ubc13 were calculated, (4.4×10^−12^ M for compound Ia and 4.68×10^−7^ M for compound IIa). (**D**) Effect of compound Ia on *in vitro* wild-type ubiquitin free chain elongation dependent on Ubc13 and Uev1. Purified recombinant proteins (E1, Ubc13, Uev1) were used to synthesize free ubiquitin chains for the indicated times, in the absence (Control) or presence of compound Ia (1 µM). Ubiquitin chains were detected by Western blotting with anti-ubiquitin. (**E**) Graphic representation of the kinetic experiment shown in (D), with quantitation of the accumulated levels of triubiquitin (Ub_3_) free chains. (**F**) Effect of compound Ia on *in vitro* chain elongation of K63- or K48-only ubiquitin mutants. The experiment was performed as in (D), except that mutant ubiquitins were substituted for the wild-type form.

Two cyclic compounds were synthesized on the basis of the structures selected from the virtual screening, and designated hereafter Ia (family I) and IIa (family II) (Fig. 2C and 2D). Both compounds interfered with the Ubc13-Uev1 interaction at micromolar concentrations on yeast two-hybrid assays (Fig. S1). In competition assays with recombinant proteins, compound Ia inhibited the Ubc13-Uev1 interaction at nanomolar concentrations, and compound IIa at micromolar concentrations (Fig. 3A). These actitivies were specific to these two compounds, since an unrelated control cyclic compound with a similar ring structure (of the family I type) did not detectably interfere with the Ubc13-Uev1 interaction at the same concentrations (Fig. 3A). This activity was quantitated by surface plasmon resonance (SPR). With this technique, the dissociation constant for the Ubc13-Uev1 interaction was 1.0×10^−9^ M, indicating a high-affinity binding of the heterodimer, with values close to those reported by isothermal titration calorimetry [40] that are expected to require high affinity binding by any potential competitor. SPR determinations yielded a IC_50_ of 1.0×10^−11^ M for compound Ia, and of 1.1×10^−6^ M for compound IIa (Fig. 3B), indicating a significantly more effective inhibition of the Ubc13-Uev1 interaction by compound Ia. They also indicated that the binding of the two active compounds on Ubc13 must occur at high affinities, in order to successfully compete with the high affinity Ubc13-Uev1 interaction. To determine the binding efficiency of these compounds to Ubc13, lysine-conjugated derivatives (Fig. S2) were immobilized on SPR sensor chips, and Ubc13 subsequently applied in the mobile phase. These assays yielded dissociation constants for Ubc13 of 4.4×10^−12^ M for compound Ia and of 4.68×10^−7^ M for compound IIa (Figure 3C). This low dissociation constant for compound Ia reinforces the conclusion that it specifically occupies with high affinity the Ubc13 interface normally used to interact with Uev1, and that this is the likely mechanism by which it antagonizes this interaction.

Next, the ability of compound Ia to affect the enzymatic activity of Ubc13-Uev1 was tested in polyubiquitin chain extension reactions with defined components. In these reactions, the substrate was either wild-type ubiquitin or a variant ubiquitin in which all Lys residues, except at positions 63 or 48, were mutated to Arg (UbK63 or UbK48). The Ubc13-Uev1 dimer, but not either protein alone, supported the formation of free polyubiquitin chains of increasing lengths with either form of ubiquitin as a substrate (Fig. 3D) with robust kinetics (Fig. 3E). Addition of compound Ia strongly inhibited this reaction (Fig. 3D and E). In control experiments, the UbK48 mutant was not a substrate for free polyubiquitin chain extension by Ubc13-Uev1 (Fig. 3F), which supports the specificity of the Ubc13-Uev1 heterodimer for K63-only ubiquitin. Neither compound affected the *in vitro* Ubc4/5-dependent polyubiquitylation of proteasomal components (Fig. S3), supporting that they specifically target Ubc13 and not other E2 enzymes with canonical (K48) polyubiquitylation activities. Therefore, compound Ia is a potent disruptor of the interaction between Ubc13 and Uev1, and an effective inhibitor of K63 polyubiquitylation catalyzed by Ubc13-Uev1.

### Effects of compound Ia on cellular pathways regulated by K63 polyubiquitylation

**Figure 4:**
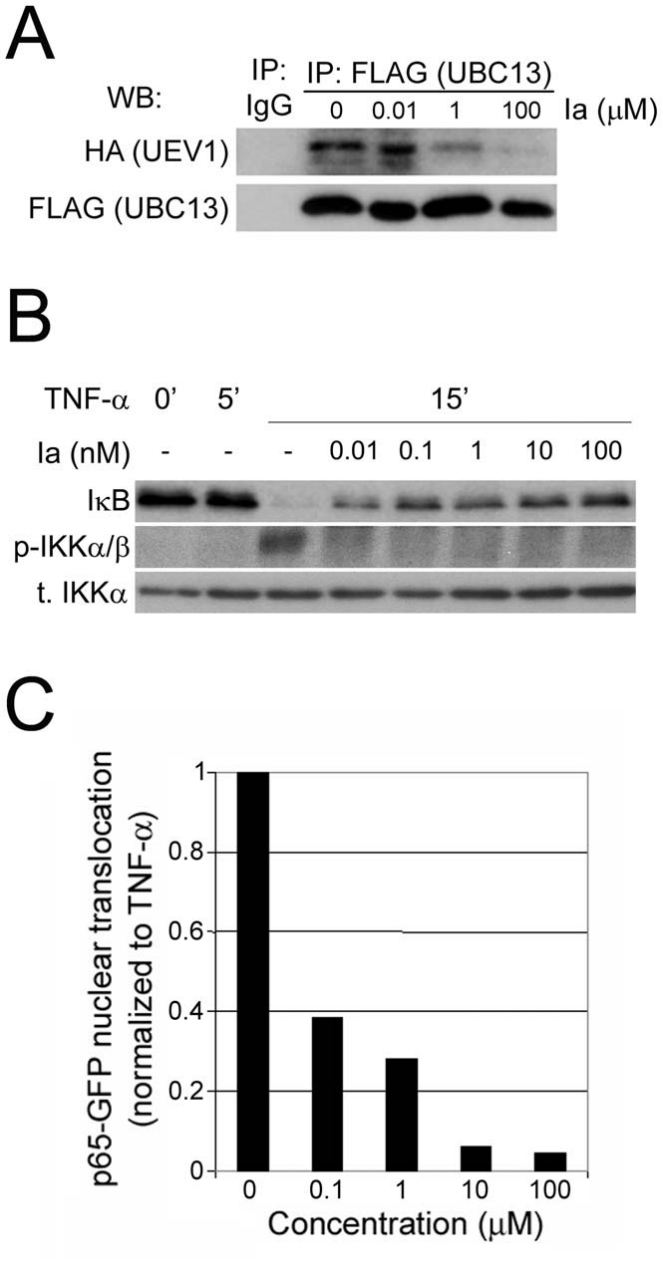
Inhibition of NF-κB signalling by compound Ia. (**A**) Compound Ia inhibits the formation of Ubc13-Uev1 heterodimers *in vivo*. HeLa cells were co-transfected with FLAG-Ubc13 and HA-Uev1, treated with different concentrations of compound Ia, lysed and subjected to co-immunoprecipitation. It can be seen that incubation of cells with increasing concentrations of compound Ia leads to decreasing amounts of Uev1 that can be co-immunoprecipitated with Ubc13. (**B**) Dose-dependent inhibition by compound Ia of NF-κB activation by TNF-α. HeLa cells were stimulated for 15 min with TNF-α (20 ng/mL) without (0) or with overnight preincubation with the indicated final concentrations of compound Ia, and analyzed by Western blotting for IκB protein levels or for phosphorylation of IKKα/β. (**C**) Dose-dependent inhibition by compound Ia of the nuclear translocation of p65-GFP induced by TNF-α. HeLa cells were transfected with p65-GFP were incubated overnight with varying concentrations of compound Ia, treated with TNF-α (20 ng/mL) for 30 min, fixed and cells counted for nuclear localization of p65-GFP. Background (proportion of cells with nuclear p65-GFP in untreated samples) was subtracted, and values normalized against the proportion of cells with nuclear p65-GFP in cells stimulated with TNF-α only.

Both compound Ia and compound IIa were shown to efficiently enter cultured human cells, as determined by HPLC of cytosolic lysates (not shown) and by microscopic localization of fluoresceinated derivatives of the compounds (Fig. S4 and S5). To test whether compound Ia could disrupt the Ubc13-Uev1 interaction *in vivo*, HeLa cells were incubated with compound Ia, lysed and subjected to co-immunoprecipitation. It can be seen (Fig. 4A) that incubation with compound Ia significantly inhibited the abundance of the Ubc13-Uev1 heterodimer in HeLa cells.

The activation of NF-κB by extracellular signals is regulated by Ubc13-dependent K63 polyubiquitylation in HEK293T and other epithelial cells [41], but not in immune cells [24]. Preincubation of HeLa cells with compound Ia prevented the downregulation of IκB by TNF-α in a dose-dependent manner, with strong effects at concentrations as low as 10 nM (Fig. 4B), an evidence of its activity as an antagonist of NF-κB activation induced by TNF-α in these cells. Compound Ia also inhibited the phosphorylation of IKKα/b induced by TNF-α in HeLa cells (Fig. 4B), consistent with the known role of Ubc13 in TNF-α signaling upstream from the IκB kinases [41]. In addition, compound Ia also strongly inhibited in a dose-dependent manner the nuclear translocation of a GFP form of p65 (a subunit of NF-κB) by TNF-α in HeLa cells (Fig. 4C).

**Figure 5:**
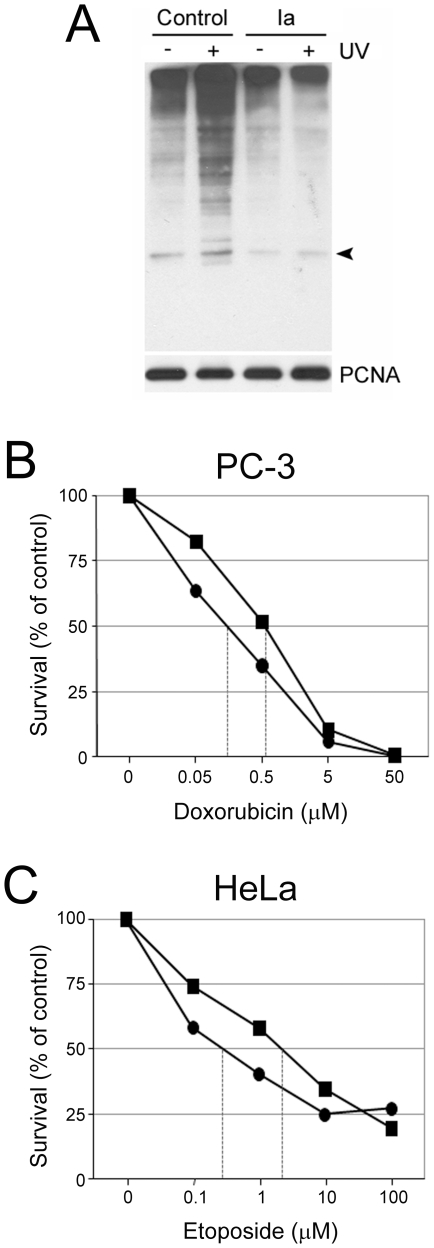
Chemosensitizing activity of compound Ia. (**A**) Inhibition by compound Ia of UV-induced K63-dependent polyubiquitylation of PCNA. HEK-293T cells, transfected with a construct expressing HA-tagged K63-only mutant ubiquitin, were pretreated (Ia) or not (Control) overnight with 100 µM of compound Ia, irradiated or not with UV light (60 J/m^2^), lysates immunoprecipitated with anti-PCNA and blotted with anti-HA. The immunoprecipitates were blotted in parallel with anti-PCNA for loading control (PCNA, lower panel). Arrowhead, position cooresponding to monoubiquitylated PCNA. (**B and C**) Sensitization by compound Ia to doxorubicin-induced cytotoxicity in mammalian cells. PC-3 prostate cancer cells (**B**) and HeLa cervical cancer cells (**C**) were exposed to varying concentrations of doxorubicin or etoposide, with or withoud compound Ia (100 µM). Viable cell numbers were determined with the CyQuant assay after 72 h (PC-3 cells) or 48 h (HeLa cells) of treatment.

We tested the effect of compound Ia on a second pathway regulated by K63-linked polyubiquitylation. Thus, compound Ia inhibited the UV-induced K63 polyubiquitylation of PCNA *in vivo* (Fig. 5A), although the effects were evident only when cells were exposed to relatively high concentrations of the compound (100 µM). In control experiments, the UV-induced K63-dependent polyubiquitylation of PCNA was inhibited by transfection with a dominant-negative form of Ubc13 (Ubc13^C87A^), indicating that this modification of PCNA indeed requires enzymatically active Ubc13 (Fig. S6).

### Chemosensitization of mammalian cells by a compound Ia

Given the role of K63 polyubiquitylation in DNA damage recognition and repair and NF-κB signaling, we reasoned that mammalian cells exposed to compound Ia might show increased sensitivities (decreased survival) to DNA damaging agents. PC-3 prostate cancer cells and HeLa cells were exposed to increasing doses of the topoisomerase II inhibitors etoposide or doxorubicin, respectively, which induce double-strand breaks in DNA, in the presence of compound Ia. In PC-3 cells, treatment with 100 µM compound Ia decreased the LD_50_ for doxorubicin from 0.55 µM to 0.1 µM (5.5-fold sensitization, Fig. 5B), and in HeLa cells, compound Ia decreased the LD_50_ for etoposide from 2.5 µM to 0.2 µM (12.5-fold sensitization, Fig. 5C).

### Effects of compound Ia on the growth and invasiveness of cultured cells

**Figure 6:**
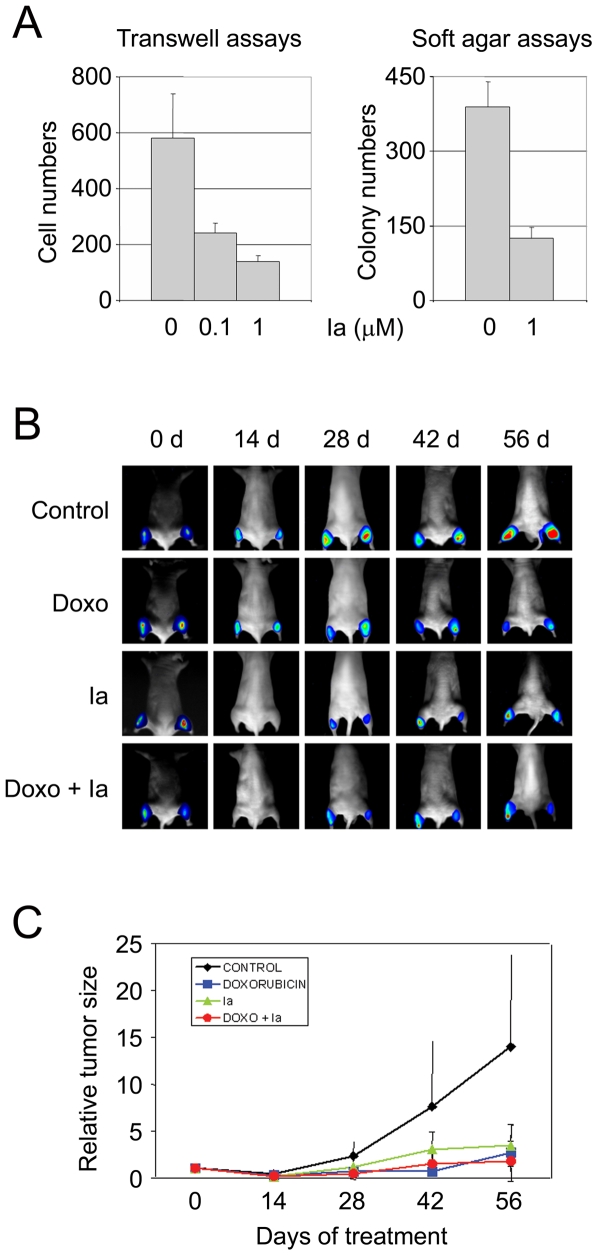
Effect of compound Ia on the growth and tumorigenicity of PC-3 prostate cancer cells. (**A**) Inhibition by compound Ia of the invasiveness through Matrigel of PC-3/luc cells (left panel) and of the clonogenicity in soft agar of PC-3/M cells (right panel). Error bars correspond to standard deviations for values obtained in triplicate experiments. (**B**) Activity of compound Ia in a mouse tumor xenograft model. Balb/c nu/nu mice were grafted with luminescent PC-3/luc cells on both rear extremities. After tumors had developed at the grafted sites, mice were placed in 4 groups. A control group received only intratumoural injections of PBS. A group was treated intravenously with doxorubicin (5 mg/Kg/week). A third group was treated with compound Ia, (100 µM intratumoral, twice a week). A fourth group was treated both with doxorubicin (5 mg/Kg/week) and compound Ia (0.15 mg/Kg intratumoral, twice a week). (**C**) Quantitative data analysis (relative light units) corresponding to 6 tumors (3 mice) for each treatment group. RLUs were normalized in each group relative to values for day 0.

Compound Ia did not significantly inhibit the growth on plastic substrate of HeLa cells under standard growth conditions (Fig. S7), suggesting a low direct cytotoxicity on a range of cell types. In contrast, it significantly inhibited colony formation in soft agar of highly metastatic and clonogenic PC-3/M cells at low micromolar concentrations (Fig. 6A, left panel), and it inhibited the *in vitro* invasiveness through extracellular matrix of parental PC-3 prostate cancer cells also at low micromolar concentrations (Fig. 6A, right panel). Neither compound Ia, nor the less active compound IIa, significantly affected the growth or differentiation of human endothelial cells *in vitro* (data not shown).

### Antitumoral activity of compound Ia in a mouse xenograft model

The chemosensitizing effect of compound Ia could be caused either by an inhibitory activity on DNA repair, a diminished activation of NF-κB, or a combination of both activities. Compound Ia also inhibits the clonogenicity and invasiveness of PC-3 cells. We next tested the *in vivo* antitumoral activity of this compound. PC-3 prostate cancer cells, bearing a stably integrated luciferase gene under the control of a constitutive promoter [42], were grafted into the rear extremities of Balb/c nude mice. Tumor-bearing mice were injected intravenously with doxorubicin (5 mg/Kg, weekly), combined, or not, with intratumoral injections of compound Ia (0.15 mg/Kg in phosphate-buffered saline, twice a week). A third group of mice received intratumoral compound Ia and no doxorubicin. Control mice were sham injected with phosphate-buffered saline by both routes. After 8 weeks of treatment, control mice had grown extensive tumors at the site of grafting (Fig. 6B, C). In contrast, mice receiving doxorubicin alone, compound Ia alone, or a combined treatment of systemic doxorubicin and local (intratumoral) compound Ia had significantly reduced tumors as compared to control mice, by the end of the treatment (Fig. 6B, C). Analysis of the corresponding quantitative data (normalized photon counts) showed that the average sizes of the tumors at the end of the experiment were 24.7% of control tumors for mice treated with compound Ia alone, 22.0% for mice treated with doxorubicin alone, and 15.5% for mice with the combined treatment (Fig. 6C), the differences between the three treatment regimes not being statistically significant. There were no evidences of acute or chronic toxicity either at the site of injection or in other organs and tissues associated with the administration of compound Ia (unpublished observations). Therefore, administration of compound Ia alone significantly limits the growth of PC-3 prostate cancer xenografts, to a degree comparable to treatment with the chemotherapeutic agent doxorubicin.

## Discussion

In terms of drug design, the specific contribution of our approach has been to use *in vivo* screenings to find hit structures from a combinatorial chemical library, and to further select the best ligands by virtual screening. Our drug development scheme should be applicable to the design of small molecules capable of specifically interfering with many other well-characterized inter- or intra-molecular interactions with amenable surfaces [31]. Other non-peptide small molecules that disrupt specific protein-protein interactions have been successfully developed in recent times [43], [44], [45], [46], [47], and they are beginning to show great promise for the therapy of human cancer. In practical terms, we have developed small molecules that can effectively and selectively antagonize the Ubc13-Uev1 interaction and inhibit K63 polyubiquitylation in both yeast and mammalian cells, and we have shown that these compounds can be used in combination therapy schemes as antitumoral agents in cultured and animal models of cancer.

Specifically, compound Ia sensitizes PC-3 prostate cancer cells to the antiproliferative activity of doxorubicin in cultured cells and it shows direct antitumoral activity in mouse tumor xenografts. A number of mechanisms can be at play to cause increased sensitivities of tumor cells to chemotherapy or radiotherapy, including inhibition of NF-κB [48], downregulation of transporters of the MDR family [49] or the Akt-mTOR pathway [50], [51], [52]. The evidence provided here suggests that at least two mechanisms may be relevant for the increased sensitivity to doxorubicin caused by compound Ia, namely inhibition of NFk-B activity and compromise of DNA repair. The demonstration that this compound disrupts the interaction between Uev1 and Ubc13 provides a mechanistic explanation for its inhibitory activity on the NF-κB signaling pathway [17]. Recently, it has been shown that another ubiquitin conjugating enzyme, UbcH5, can promote K63 polyubiquitylation, and that NF-κB activation by IL-1β is much more strongly dependent on Ubc13-dependent K63 polyubiquitylation than activation by TNF-α [22]. However, a large body of literature strongly suggests a critical role of Ubc13 and K63 polyubiquitylation in the activation of NF-κB not only by IL-1β but also by TNF-α. In this regard, the chain type of ligand-induced ubiquitylation by cIAP of TNF-R1 complex components has not been determined, and, given the recruitment of Ubc13 by cIAP, it is quite possible that such chains are of the K63 type. Furthermore, mice haploinsuficient for Ubc13 display cell-type-specific defects in chemokine and NF-κB signaling [53], supporting a critical role of Ubc13 and K63 polyubiquitylation in the activation of NF-κB by different stimuli *in vivo*, including TNF-α and LPS. Our observations showing that the small molecule antagonist of Ubc13-Uev interactions compound Ia inhibits NF-κB activation by TNF-α would also support a role for Ubc13 in this pathway. Alternative explanations would include the possibility that our compounds inhibit other ubiquitin conjugating enzymes or additional components of the TNF-α signaling cascade, which has not been formally ruled out in the present study.

On the other hand, it has also been shown that unanchored K63-linked polyubiquitin chains are essential for the activation of the RIG-I pathway in response to viral infection, and that both Ubc13 and Ubc5 are required in this pathway [54], [55]. Therefore, the inhibition of Ubc13 by small compounds could limit the response to viral infections mediated through this pathway.

Regarding the role of Ubc13 and K63 polyubiquitylation in DNA damage response, the very high similarity of Uev2 (Mms2) to Uev1, and the computed interaction of compound Ia on the hydrophobic pocket of Ubc13, allows to predict with sufficient confidence that this compound should disrupt also the interaction of Uev2 (Mms2) with Ubc13. Indeed, we have shown that compound Ia inhibits the UV-induced K63 polyubiquitylation of PCNA, a modification that requires Ubc13-Uev2 [11], [15], [16]. Therefore, the predicted disruption of the Ubc13-Uev2 heterodimer should be associated with a compromise in tolerance to DNA damage by radiation or radiomimetic drugs in mammalian cells [16]. Additional mechanisms, not explored here but possibly also involved in the chemosensitization caused by compound Ia, could be related to the regulation by Ubc13 of double-strand DNA damage recognition and repair through its interaction with the ubiquitin ligase RNF8 [12]. The fact that we have observed inhibition by compound Ia of K63 polyubiquitylation of PCNA only at high concentrations of the compound may suggest either that the compound, although it enters the cells, does not reach the nucleus efficiently, or that K63 polyubiquitylation of PCNA can be catalyzed in mammalian cells by other ubiquitin conjugating enzymes in addition to Ubc13. This may also be the case for K63 polyubiquitylation associated with damage foci in response to DNA double-strand breaks [56]. Indeed, in immunofluorescent γ-H2AX focus assays, the same batches of compound Ia that inhibited NF-κB activation at low micromolar concentrations only modestly inhibited the maintenance of γ-H2AX in ionizing radiation-induced foci (data not shown). Given the limited effects of compound Ia on both PCNA K63-linked polyubiquitylation and on DNA damage focus formation and resolution, it is possible that the chemosensitization to doxorubicin and etoposide observed in PC-3 and HeLa cells may be better explained by its inhibitory effects on NF-κB signaling.

We have observed that compound Ia exerts a direct antitumoral activity in a PC-3 mouse xenograft tumor model. This compound was not directly antiproliferative *in vitro* for a variety of cell lines tested, but it inhibited the invasiveness of PC-3 cells through extracellular matrix in Boyden chamber experiments, and also inhibited the formation of colonies in 3-dimensional soft-agar cultures. The NF-κB pathway is known to play a prominent role in promoting invasiveness [57], [58], being constitutively active in PC-3 cells [59], and thus the observed inhibition of *in vitro* invasiveness by compound Ia could be one of the consequences of the inhibition of NF-κB activation by this compound. Clonogenicity in soft agar is associated with the capacity of cells for self-renewal, and tends to correlate well with tumorigenicity *in vivo*. This property, exhibited by distinct cellular subpopulations in some tumors, is not necessarily positively correlated with NF-κB activity [60], and thus the inhibition by compound Ia of the clonogenicity of PC-3 cells could reflect a requirement for Ubc13 activity in other pathways regulating the self-renewal capacity of these cells. In any case, the sum of both activities of compound Ia could explain at least part of the observed direct antitumoral effect.

In summary, we have developed specific and potent small molecule antagonists of the Ubc13-Uev1 interaction that inhibit the enzymatic activity of this heterodimer, K63 polyubiquitylation, and we have shown that one of these molecules produces significant effects in the activation of NF-κB by TNF-α, and in invasiveness and clonogenicity *in vitro* and tumorigenicity of cancer cells *in vivo*. Based on these activities, we anticipate that tese compounds should be useful to probe other biochemical pathways and cellular processes regulated by K63 polyubiquitylation and to test their effects in relevant models of human pathologies in which these processes are dysregulated.

## Materials and Methods

### Synthesis of trialkylglycine-based combinatorial mixtures and of individual compounds

An optimized library of 5,120 peptoids in 52 controlled mixtures was synthesized by using the positional scanning format on solid phase [33] (Supporting Methods S1).

### Yeast two-hybrid

Plasmids pBD-Ubc13 and pACT2-Uev1 were co-transfected into the S. cerevisiae strain AH109 in quadruple selection medium. After 3 days, colonies positive for the Ubc13-Uev1 interaction were subjected to liquid culture assays of β-galactosidase activity using ONPG as the substrate, in the presence of 0.1 mM of peptoid-mix from each peptoid-pool. One unit of β-galactosidase was defined as the amount which hydrolyzed 1 µmol O-nitrophenol D-galactose per min per cell.

### Docking

Docking was performed using the crystal structure of the Ubc13-Mms2 complex (entry 1JAT in the Brookhaven Protein Data Bank). From the heterodimeric complex, chain A (Ubc13) was selected as the receptor (Supporting Methods S1).

### Protein-protein binding assays

Purified GST-Ubc13 (1 µg) was bound to glutathione-Sepharose, washed and preincubated for 1 h with varying concentrations of compounds Ia IIa, or a control cyclic compound, and then allowed to bind Uev1 (1 µg) for 1 h in a buffer containing 50 mM Tris-HCl pH 7.6, 0.1 mM EDTA, 150 mM NaCl and 0.2 mM DTT. Unbound fractions were collected, the beads washed 5 times with the same buffer, and finally incubated with glutathione elution buffer. Fractions were resolved by SDS-PAGE, and Ubc13 and Uev1 detected by Western blotting with specific antibodies [61].

### Surface plasmon resonance

Recombinant Uev1 protein was immobilized on a Biacore CM5 Chip. The final response obtained was 1179,1 RU (response units) (1 response unit corresponds to 1 pg of protein per mm^2^) of immobilized Uev1. Purified Ubc13 was maintained at 400 nM in running buffer (10 mM HEPES, 0.15 M NaCl, pH 7.4). Ubc13 was preincubated with the test compounds at varying concentrations, and interaction assays run in a Biacore T100 instrument.

### *In vitro* ubiquitylation

Purified Ubc13 (0.2 µM), Uev1 (0.2 µM), and E1 (0.1 µM, Boston Biochem) were incubated in 50 mM Tris-HCl, 5 mM MgCl_2_, 2 mM ATP and 0.5 mM DTT, with either 117 µM wild-type ubiquitin (Biomol), 6 µM UbK48_only or 6 µM UbK63_only (Boston Biochem). To determine the effects of compounds Ia and IIa, Ubc13 was pre-incubated for 10 min at room temperature with 1–100 µM of either compound. The reactions were allowed to proceed for the indicated times and stopped by addition of SDS-PAGE sample buffer and boiling. The reaction products were detected by Western blotting with a rabbit anti-ubiquitin antibody (Biomol).

### Cell culture

HeLa and HEK293T cells were obtained from the American Type Culture Collection (Manassas, VA). PC-3/M cells [62] were kindly provided by Dr. Isaiah Findler, and PC-3/luc cells were described in [42]. All cells were grown in an atmosphere of 5% CO_2_, 95% humidity at 37°C in RPMI 1640 or DMEM supplemented with 10% fetal bovine serum, 1 mM sodium pyruvate, 100 U/mL penicillin, 100 µg/mL streptomycin, 2 mM L-glutamine and 0.1 mM non-essential aminoacids (PAA, Pasching, Austria). PC-3/luc cells were grown in complete medium supplemented with 200 µg/mL Geneticin (Sigma, Alcobendas, Spain) to maintain integrated copies of the luciferase gene.

### Chemosensitization assays in mammalian cells

HeLa and PC-3 cells were treated with varying concentrations of doxorubicin or etoposide, together, or not, with 100 µM of compound Ia. Viable cell numbers were determined at 24, 48 and 72 hours with the CyQuant assay.

### *In vivo* ubiquitylation of PCNA

HEK293T cells, transfected with pcDNA-HA-UbK63, were incubated overnight with 100 µM of compound Ia, followed by UV irradiation (60 J/m^2^). After 6 h, cells were harvested, immunoprecipitated with anti-PCNA (Santa Cruz Biotechnology, Santa Cruz, CA), immune complexes resolved by SDS-PAGE and immunoblotted with anti-HA rat monoclonal antibody (Roche, Mannheim, Germany). As a loading control, immunoprecipitates were immunoblotted with anti-PCNA.

### NF-κB assays

To determine IκB protein levels, HeLa cells were incubated overnight with compound Ia, and treated with TNF-α (20 ng/mL) for 15 min. Cells were lysed and analyzed by Western blotting with anti-IκB (Cell Signaling, Danvers, MA). To analyze IKKα/β phosphorylation, the same lysates as above were tested by Western blotting with an antibody to phospho-IKKα (Ser180)/β (Ser181), or to total IKKα (Cell Signaling). For nuclear translocation assays, HeLa cells grown on sterile coverslips were transfected with pPCR3/p65-GFP, incubated overnight with varying concentrations of compound Ia, and treated with TNF-α (20 ng/mL) for 30 min. Samples were fixed, counterstained with Hoechst 33258, and images captured with a fluorescence microscope. At least 3 coverslips and 200 cells from 10 separate fields were counted and scored for positive nuclear green flourescence. The proportion of cells with nuclear p65-GFP in untreated cells was taken as background. Background subtracted values were normalized against those for cells treated with TNF-α alone.

### Invasiveness assays

PC-3/luc cells (5×10^4^), preincubated for 24 h with compound Ia, were resuspended in 0.1% BSA and seeded on 12-well, 8-µM pore diameter Transwell chambers (Costar, Cambridge, MA) coated with diluted (1∶20 in H_2_O) growth factor-reduced Matrigel (Beckton-Dickinson, San Jose, CA). After 24 h of incubation in the presence of compound Ia in both chambers, cells in the top chamber were removed, cells in the lower chamber were assayed for luciferase activity, and cell numbers inferred from standard luciferase-activity curves. Each condition was assayed in triplicate.

### Soft agar colony formation

Melted agar (0.5%) in complete medium was placed at the bottom of 12-well plates, allowed to solidify and overlayed with 3×10^3^ PC-3/M cells resuspended in 0.3% agar/complete medium. Cells were fed three times a week with medium containing compound Ia. After 2 weeks, wells were fixed with 0.5% glutaraldehyde, stained with 0.025% crystal violet, images captured and analyzed with the Image J software. Only colonies ≥0.1 mm diameter were scored. Each condition was assayed in triplicate.

### Tumor xenograft analyses

PC-3/luc cells (5×10^5^) were injected into each rear limb of male Balb/c nu/nu mice. Tumor size was assessed by photon counts in a ORCA-2BT instrument (Hamamatsu Photonics, Hamamatsu City, Japan). Luciferin (16.7 mg/ml in PBS) (Promega) was injected intraperitoneally into anestesized mice, and images acquired for 5 min and quantitated. Light measurements were expressed as photon counts (PHCs), and values normalized vs. initial counts for each tumor.

## Supporting Information

Supporting Methods S1 (0.07 MB DOC)

Table S1 (0.03 MB PDF)

Table S2 (0.03 MB PDF)

Figure S1 Antagonism by compounds Ia and IIa of the Ubc13-Uev1 interaction, assayed by yeast two-hybrid. Yeast cells (AH109 strain), co-transfected with pBD-Ubc13 and pACT2-Uev1 and grown in quadruple selection medium, were incubated overnight with 100 µM of cyclic compounds Ia or IIa, and assayed for α-galactosidase activity as a semiquantitative measure of strength of interaction. Values were normalized against those of cells incubated with a control, unrelated cyclic compound. As an additional control, yeast cells harboring large T and p53 were assayed in parallel under identical treatments and conditions.
(1.17 MB TIF)

Figure S2 Structures of lysine derivatives of cyclic compounds Ia (A) and IIa (B). The two compounds were synthesized with a lysine residue covalently attached to their free amide groups, as described in Supporting Methods S1.
(0.26 MB TIF)

Figure S3 Compound Ia does not inhibit Ubc4-dependent polyubiquitylation of proteasome-associated components. Components of the proteasome holoenzyme undergo K48-based polyubiquitylation in the presence of the ubiquitin ligase Hul5 and the E2 enzyme Ubc4 in a 1-h reaction. Compound Ia does not inhibit the formation of Ubc4-dependent high molecular weight ubiquitin adducts at any of the concentrations tested. DMSO denotes the addition of the solvent at the concentration equivalent to that added when using the maximum concentration of compound Ia used in this experiment (500 µM).
(1.42 MB TIF)

Figure S4 Structures of fluoresceinated derivatives of cyclic compounds Ia (A) and IIa (B). The two compounds were synthesized with a fluoresceine isotiocyanate moiety covalently attached to their free amide groups, as described in Supporting Methods.
(0.40 MB TIF)

Figure S5 Uptake by mammalian cells of fluoresceinated compounds Ia (Ia-FITC) and IIa (IIa-FITC). HeLa cells, grown on sterile coverslips, were incubated overnight with 100 µM of either Ia-FITC or IIa-FITC, and processed for immunocytochemistry for detection of Ubc13. As a control, HeLa cells were incubated with unconjugated FITC.
(4.19 MB TIF)

Figure S6 UV-induced, K63-type polyubiquitylation requires enzymatically active Ubc13. PCNA undergoes K63-based polyubiquitylation upon UV irradiation, which is inhibited by transfection of a dominant-negative form of Ubc13 (Ubc13C87A). HeLa cells were transfected with HA-UbK63, together, or not, with pcDNA3.1-Ubc13C87A. After a 24-h preincubation with compound Ia (1 µM), cells were exposed, or not, to UV radiation (60 J/m^2^), lysed, immunoprecipitated with anti-PCNA, and K63-based polyubuiquitin chains detected by Western blotting with anti-HA.
(0.65 MB TIF)

Figure S7 Growth curves of HeLa cells incubated with cyclic compounds Ia (top) or IIa (bottom). Cells were grown for up to 4 days in the presence of varying concentrations of either cyclic compound, freshly added every 48 h, and cell numbers determined by the CyQuant procedure. Shown are average values for each time point and treatment condition, which were done in octuplicate.
(7.72 MB TIF)
