# Supplementary material for: Protein-Protein Interaction Antagonists as Novel Inhibitors of Non-Canonical Polyubiquitylation
Source: PLoS One. 2010 Jun 30;5(6):e11403. doi: 10.1371/journal.pone.0011403 (PMC2894972; doi:10.1371/journal.pone.0011403)
Supplement: Supporting Methods S1 — (0.07 MB DOC) [file pone.0011403.s001.doc]

**Supporting Information**

**Protein-protein interaction antagonists as novel inhibitors of non-canonical polyubiquitylation**

Johanna Scheper, Marta Guerra-Rebollo, Glòria Sanclimens, Alejandra Moure, Isabel Masip, Domingo González-Ruiz, Núria Rubio, Bernat Crosas, Óscar Meca-Cortés, Noureddine Loukili, Vanessa Plans, Antonio Morreale, Jerónimo Blanco, Angel R. Ortiz, Àngel Messeguer, and Timothy M. Thomson

Contains:

Supporting Methods

Supporting References

**Supporting Methods**

*Synthesis of trialkylglycine-based combinatorial mixtures and of individual compounds*. An optimized library of 5,120 peptoids in 52 controlled mixtures was synthesized by using the positional scanning format on solid phase [1]. The mixture positions were incorporated by coupling a mixture of 20 or 16 selected primary amines with the relative ratios adjusted to yield equimolar incorporation [2]. Individual peptoids were prepared by simultaneous multiple solid-phase synthesis following the following synthetic sequence: *N*37-37-9*C* [*N*-(2-(4’-fluorophenyl)ethyl)glycyl]-[*N*-(2-(4’-fluorophenyl)ethyl)glycyl]-[*N*-(2-(2’-pyridin)ethyl]glycinamide; ESI-MS: C_29_H_33_F_2_N_5_O_3_ calcd. [M+H^+^] 538.3; found: [M+H^+^] 538.2; [M+Na^+^] 560.1; *N*37-37-13*C* [*N*-(2-(4’-fluorophenyl)ethyl)glycyl]-[*N*-(2-(4’-fluorophenyl)ethyl)glycyl]-[*N*-(2-(4’-methoxyphenethyl]glycinamide; ESI-MS: C_31_H_36_F_2_N_4_O_4_ calcd. [M+H^+^] 567.3; found: [M+H^+^] 567.2; [M+Na^+^] 589.2; *N*15-37-13*C* [*N*-(2-(2’,4’-dichlorophenyl)ethyl)glycyl]-[*N*-(2-(4’-fluorophenyl)ethyl)glycyl]-[*N*-(2-(4’-methoxyphenethyl]glycinamide; ESI-MS: C_31_H_35_Cl_2_FN_4_O_4_ calcd. [M+H^+^] 617.2; found: [M+H^+^] 617.1; *N*15-37-9*C* [*N*-(2-(2’,4’-dichlorophenyl)ethyl)glycyl]-[*N*-(2-(4’-fluorophenyl)ethyl)glycyl]-[*N*-(2-(2’-pyridin)ethyl]glycinamide; ESI-MS: C_29_H_32_Cl_2_FN_5_O_3_ calcd. [M+H^+^] 588.2; found: [M+H^+^] 588.1; [M+Na^+^] 610.1.

Cyclic derivatives Ia and IIa were synthesized by solid-phase methodology and microwave-asssited reactions which will be reported in detail elsewhere. Ia: *N*-aminocarbamoylmethyl-*N*-(2’-(2’’pyridyl)ethyl) 1,4-bis[2’-(4’’-fluorophenyl)ethyl]-3,7-dioxo-[1,4]diazepane-5-carboxamide; HRMS-FAB: C_31_H_33_F_2_N_5_O_4_ calcd [M+H^+^] 578.257886; found 578.257844. IIa: [1,4-bis[2’-(4’’-fluorophenyl)ethyl]-2-[*N*-aminocarbonylmethyl-*N*-(2’-(2’’pyridyl)ethyl)carbonylmethyl]piperazine-3,6-dione, HRMS-FAB: C_31_H_33_F_2_N_5_O_4_ calcd [M+H^+^] 578.2501; found 578.2573. The purity and identity of of all compounds were determined by analytical high-performance liquid chromatography and ^1^[H] and ^13^[C] NMR.

To synthesize a fluoresceinated derivative, compound Ia was converted into its carboxylic acid derivative and then coupled to a lysine residue for the attachment of the fluorescein moiety. 5’-6’ Carboxyfluorescein-labelled Ia: ESI-MS: C_58_H_55_F_2_N_7_O_11_ calcd. [M+H^+^] 1064.39; found: [M+H^+^] 1064.3; [M+2H^+^]/2 532.8. For compound IIa, a specific intermediate, [1,4-bis[2’-(4’’-fluorophenyl)ethyl]-2-[N-(4-aminobutylcarbamoylmethyl)-N-(2’-(2’’pyridyl)ethyl)carbonylmethyl]piperazine-3,6-dione, was synthesized in solid phase and treated with carboxyfluorescein to yield the fluoresceinated derivative. 5’-6’ Carboxyfluorescein-labelled IIa: ESI-MS: C_56_H_52_F_2_N_6_O_10_ calcd. [M+H^+^] 1007.3713; found: [M+H^+^] 1007.3; [M+2H^+^]/2 504.2.

*Docking***.** Docking was performed using the crystal structure of the UBC13-MMS2 complex (entry 1JAT in the Brookhaven Protein Data Bank). From the heterodimeric complex, chain A (UBC13) was selected as the receptor. Hydrogen atoms were added using the *protonate* function from the AMBER package suite [3]. For both docking and energy calculations, the protein was described by the *parm99* AMBER force field. From the selected peptoids (ligands), structural variants were initially built with CORINA and the in-house algorithm ALFA. Charges were calculated by electrostatic potential fitting (ESP) derived with MOPAC using the MNDO Hamiltonian [4,5]. AMBER atom types were automatically assigned to the ligands, as described [6]. The resulting library of molecules were docked within the limits of a specific space created on the surface of the receptor (UBC13), defined as a grid box with a spacing of 0.5 Ǻ, created using chemical probes (present in the peptoids). The energy function used is an adaptation of the one in the AMBER package . Docking of all prepared ligands was performed with CDOCK, with exhaustive exploration for all conformers in the ligand library, by taking 0.5 Ǻ translational and 30º rotational steps. The conformations were ranked according to their interaction energies, measured within the grid box. For the 20 topmost orientations, a final correction of the interaction energy was introduced, by a binding free energy function of the form:

ΔG interaction = ΔG_VDW_ + ΔG_ELE_ + ΔG_HYD_ + ΔGCON [1]

where the van der Waals interactions (first term) are provided by CDOCK, and the second term accounts for the electrostatic binding free energy. This term was computed numerically by solving the linear Poisson equation using the finite difference method as implemented in *Delphi.* Each system was immersed in a cubic box occupying 65% of the total volume, with a 0.5 Ǻ grid spacing. The interior of the protein, ligands, and complexes were set to a low dielectric constant (ε = 4), while the exterior was treated as a high dielectric medium (ε = 80). The boundary surface separating solute from solvent was calculated using a solvent-probe radius of 1.4 Ǻ, leaving a minimum separation of 11 Ǻ between any solute atom and the borders of the box. The third term in equation [1] accounts for the hydrophobic binding free energy. We considered this term to be proportional to the solvent accessible surface lost upon complex formation. The fourth term is an entropic contribution that accounts for the penalty associated to the freezing of the ligand torsional degrees of freedom upon complex formation. This is modeled considering that in the complex the ligand is restricted to a single conformation, while in solution the molecule explores different conformations, as computed during the ligand conformational search with CDOCK.

*Assays of ubiquitin-protein ligase activity*. Incubations were performed in 25 mM Tris-HCl (pH 7.5), 1 mM DTT, 0.5 mM EDTA, 5 mM ATP buffer, in 50-μl reactions at 30 °C during 1 hour, containing 2 μg holoenzyme, 0.1 μg E1 (Boston Biochem, Cambridge, MA), 1 μg purified Hul5 (ubiquitin ligase), 0.1 μg Ubc4 (E2; Boston Biochem) and 1 μg of His_6_-tagged ubiquitin (Boston Biochem). Depending on the reaction, compound Ia was added at indicated concentrations. The formation of ubiquitylated adducts was detected by Western blotting with an anti-ubiquitin antibody (Biomol).

*Immunocytochemistry***.** Cells were grown overnight on sterile coverslips and incubated for 24 h with 100 μM of FITC derivatives of compound Ia or IIa, washed, fixed with 4% paraformaldehyde, permeabilized with permeabilization/blocking buffer (0.1 % saponine, 1 % BSA and 1/40 normal goat serum), incubated with anti-UBC13 for 2 h at room temperature in a humid chamber, washed, and incubated for 1 h with goat anti-rabbit IgG-TRITC (Sigma), washed and mounted with Immuno-Fluor Medium (ICN, Costa Mesa, CA). Confocal images were captured with a Leica TCS 4D confocal microscope.

*Co-immunoprecipitation*. HeLa cells were co-transfected with plasmids FLAG-UBC13 and HA-UEV1 [7], washed twice with PBS and lysed with cell lysis buffer (50 mM Tris HCl, pH 7.5, 100 mM NaCl, 1% Triton X-100 and protease inhibitors: 2 μg/ml aprotinin 2 μg/ml leupeptin, 50 μg/ml PMSF). Lysates were precleared for 1 hour with Sepharose and incubated with matrix-bound anti-FLAG antibody (Sigma) for 3 h at 4ºC. The matrix was washed 4 times with lysis buffer and the immune complexes eluted by boiling in Laemmli sample buffer. Samples were electrophoresed by SDS-PAGE, transferred to PVDF membranes and incubated sequentially with mouse anti-Flag M2 monoclonal antibody (Sigma) at 10 μg/ml and goat anti-mouse IgG-peroxidase (Dako) at a 1/2000 dilution, or rat anti-HA monoclonal antibody (Roche, Mannheim, Germany) at 200 ng/ml and goat anti-rat IgG-peroxidase (Dako) at a 1/2000, followed by ECL detection (Amersham).

*Ubc13-dependence of PCNA K63-based polyubiquitylation*. HeLa cells, transfected with pcDNA-HA-UbK63, with or without co-transfection with pcDNA3.1-Ubc13^C87A^ (dominant negative Ubc13 [7]), were incubated overnight with 100 μM of compound Ia, followed by UV irradiation (50 J/m^2^). After 6 h, cells were harvested, immunoprecipitated with anti-PCNA (Santa Cruz Biotechnology), immune complexes resolved by SDS-PAGE and immunoblotted with anti-HA rat monoclonal antibody (Roche).

*Proliferation assays in mammalian cells*. HeLa cells (10^4^ cells/well) were grown in Dulbecco’s Modified Eagle’s Medium (DMEM) (PAA Laboratories, Linz, Austria) supplemented with 10% fetal bovine serum and antibiotics in 5% CO_2_ and seeded on 96 well-plates. Twenty-four hours later they were incubated with varying concentracions of the test compounds. Cell numbers were determined at 24, 48, 72 and 144 hours by the CyQuant assay (Molecular Probes). Plates were read on a fluorimeter at an excitation maximum of 480 nm and emission maximum of 520 nm. Cell numbers were extrapolated from standard curves obtained in parallel determinations.

**Supporting References**

1. Abad-Merin MJ, Cortes N, Masip I, Perez-Paya E, Ferragut JA, et al. (2005) Trimers of N-alkylglycines are potent modulators of the multidrug resistance phenotype. J Pharmacol Exp Ther 313: 112-120.

2. Masip I, Cortes N, Abad MJ, Guardiola M, Perez-Paya E, et al. (2005) Design and synthesis of an optimized positional scanning library of peptoids: identification of novel multidrug resistance reversal agents. Bioorg Med Chem 13: 1923-1929.

3. Case DA, Cheatham TE, III, Darden T, Gohlke H, Luo R, et al. (2005) The Amber biomolecular simulation programs. J Computat Chem 26: 1668-1688.

4. Stewart JJ (1990) MOPAC: a semiempricial molecular orbital program. J Comput Aided Mol Des 4: 1-105.

5. Deward M, Thiel W (1977) Grond states of molecules. 38. The MNDO method. Approximations and parameters. J Am Chem Soc 99: 4899-4907.

6. Murcia M, Ortiz AR (2004) Virtual screening with flexible docking and COMBINE-based models. Application to a series of factor Xa inhibitors. J Med Chem 47: 805-820.

7. Plans V, Scheper J, Soler M, Loukili N, Okano Y, et al. (2006) The RING finger protein RNF8 recruits UBC13 for lysine 63-based self polyubiquitylation. J Cell Biochem 97:

572-582.
