# Supplementary material for: Protein-Protein Interaction Antagonists as Novel Inhibitors of Non-Canonical Polyubiquitylation
Source: PLoS One. 2010 Jun 30;5(6):e11403. doi: 10.1371/journal.pone.0011403 (PMC2894972; doi:10.1371/journal.pone.0011403)
Supplement: Table S1 — (0.03 MB PDF) [file pone.0011403.s002.pdf]

Table S1. Free binding energies for CDOCK selected enantiomers of family I cyclic compounds.

Enantiomer	VDW	ELEC_R	ELEC_L	COULOM	APOLAR	TOTAL
Ie1_1	-40.971	11.599	3.478	0.146	-6.433	-32.180
Ie1_2	-40.983	11.375	3.004	0.252	-6.218	-32.568
Ie1_3	-40.075	11.133	3.741	0.25	-6.310	-31.260
Ie1_4	-39.099	11.033	2.931	0.114	-6.242	-31.263
Ie1_5	-39.508	11.304	2.843	0.239	-6.216	-31.336
Ie1_6	-38.667	10.702	2.717	-0.256	-6.242	-31.746
Ie1_7	-38.702	11.811	2.781	-0.050	-6.324	-30.483
Ie1_8	-39.623	10.761	3.686	-0.303	-6.266	-31.745
Ie1_9	-37.217	8.590	3.305	-1.144	-5.475	-31.940
Ie1_10	-36.797	9.310	2.541	-0.766	-5.827	-31.539
Ie1_11	-38.32	11.859	2.601	-1.229	-6.061	-31.150
Ie1_12	-37.097	11.204	2.748	-0.841	-6.269	-30.255
Ie1_13	-33.536	10.944	3.528	-2.692	-5.615	-27.370
Ie1_14	-39.153	10.625	3.344	0.0273	-60.219	-31.177
Ie1_15	-34.082	10.389	3.494	-2.574	-5.662	-28.434
Ie1_16	-37.161	8.012	3.207	-1.155	-5.442	-32.539
Ie1_17	-32.884	11.115	3.730	-2.621	-5.444	-26.104
Ie1_18	-32.273	9.611	3.108	-2.902	-5.241	-27.697
Ie1_19	-37.195	8.997	2.951	-1.152	-60.514	-32.450
Ie1_20	-32.444	9.735	3.383	-2.875	-5.457	-27.658
Ie2_1	-35.733	10.507	2.564	-0.721	-5.633	-29.015
Ie2_2	-32.292	11.534	3.257	-3.736	-5.188	-26.425
Ie2_3	-31.315	9.782	3.590	-2.265	-5.667	-25.875
Ie2_4	-35.067	9.280	2.708	-0.356	-5.975	-29.409
Ie2_5	-35.56	9.268	3.913	-0.991	-5.356	-28.725
Ie2_6	-29.528	7.788	3.298	-3.028	-5.483	-26.954
Ie2_7	-29.447	8.242	3.131	-3.092	-5.382	-26.548
Ie2_8	-29.419	8.590	3.672	-4.084	-5.405	-26.646
Ie2_9	-33.2	9.923	3.381	-1.259	-5.440	-26.594
Ie2_10	-27.393	7.133	2.815	-3.848	-5.032	-26.325
Ie2_11	-35.041	9.015	4.100	-0.966	-5.563	-28.455
Ie2_12	-34.947	8.932	3.348	-0.587	-5.579	-28.831
Ie2_13	-27.378	6.714	2.777	-3.574	-4.883	-26.344
Ie2_14	-35.155	8.271	3.964	-0.827	-5.467	-29.213
Ie2_15	-34.24	8.083	4.238	-2.554	-5.383	-29.856
Ie2_16	-32.075	10.832	3.476	-3.245	-5.070	-26.082
Ie2_17	-35.029	9.811	3.857	-2	-5.565	-28.925
Ie2_18	-27.485	7.392	3.759	-5.067	-5.152	-26.552
Ie2_19	-31.431	10.177	3.234	-1.322	-5.384	-24.726
Ie2_20	-35.44	9.086	2.780	0.0718	-5.774	-29.276

VDW: van der Waals contribution (see Supporting Methods)
ELEC_R: Electrostatic contribution (Receptor)
ELEC_L: Electrostatic contribution (Ligand)
COULOM: Coulombic contribution
APOLAR: Apolar contribution
