# Supplementary material for: Protein-Protein Interaction Antagonists as Novel Inhibitors of Non-Canonical Polyubiquitylation
Source: PLoS One. 2010 Jun 30;5(6):e11403. doi: 10.1371/journal.pone.0011403 (PMC2894972; doi:10.1371/journal.pone.0011403)
Supplement: Table S2 — (0.03 MB PDF) [file pone.0011403.s003.pdf]

Table S2. Free binding energies for CDOCK selected enantiomers of family II cyclic compounds.

Enantiomer	VDW	ELEC_R	ELEC_L	COULOM	APOLAR	TOTAL
IIe1_1	-41.374	9.617	2.810	-0.351	-6.657	-35.954
IIe1_2	-34.3	8.844	3.626	-3.132	-5.755	-30.717
IIe1_3	-33.964	8.714	3.277	-3.381	-5.891	-31.245
IIe1_4	-33.311	10.19	3.699	-4.259	-6.348	-30.023
IIe1_5	-33.175	9.221	3.311	-3.808	-5.929	-30.380
IIe1_6	-33.923	8.732	3.202	-2.731	-5.812	-30.531
IIe1_7	-36.727	8.242	3.902	-1.684	-5.860	-32.127
IIe1_8	-39.854	8.277	2.777	-0.586	-6.332	-35.717
IIe1_9	-32.324	8.401	3.276	-3.094	-5.588	-29.329
IIe1_10	-34.224	9.982	3.435	-2.676	-5.910	-29.392
IIe1_11	-33.214	10.08	3.114	-3.267	-5.949	-29.228
IIe1_12	-31.835	8.012	3.322	-3.597	-5.758	-29.856
IIe1_13	-32.641	9.906	3.284	-3.299	-5.980	-28.730
IIe1_14	-33.542	8.944	3.019	-2.520	-5.808	-29.906
IIe1_15	-31.405	9.953	3.495	-4.360	-6.248	-28.565
IIe1_16	-32.657	8.649	3.084	-2.969	-5.455	-29.348
IIe1_17	-32.968	8.572	3.049	-2.676	-5.528	-29.550
IIe1_18	-35.642	7.829	3.494	-1.694	-5.746	-31.758
IIe1_19	-31.397	8.926	3.220	-4.340	-6.059	-29.648
IIe1_20	-32.246	8.236	3.232	-2.916	-5.512	-29.206
IIe2_1	-42.093	10.690	3.118	0.719	-6.644	-34.208
IIe2_2	-38.655	11.233	3.066	-0.351	-6.398	-31.104
IIe2_3	-37.433	10.478	2.527	0.263	-6.113	-30.277
IIe2_4	-35.582	10.018	2.874	-0.757	-6.149	-29.596
IIe2_5	-38.408	8.195	2.641	0.412	-6.187	-33.346
IIe2_6	-33.583	9.038	3.266	-0.954	-5.873	-28.104
IIe2_7	-36.464	10.897	3.215	-0.0821	-6.452	-28.885
IIe2_8	-36.416	11.038	2.956	0.206	-6.386	-28.600
IIe2_9	-33.992	10.437	2.975	-0.740	-5.797	-27.116
IIe2_10	-36.924	11.121	3.023	0.242	-6.399	-28.935
IIe2_11	-29.899	7.811	2.760	-2.630	-5.072	-27.029
IIe2_12	-38.441	8.702	2.758	0.485	-6.343	-32.838
IIe2_13	-33.03	9.463	3.036	-1.231	-5.740	-27.502
IIe2_14	-37.198	10.561	2.954	-0.903	-5.968	-30.554
IIe2_15	-32.242	10.384	2.693	-2.062	-5.464	-26.690
IIe2_16	-35.59	9.764	2.287	0.0679	-5.935	-29.406
IIe2_17	-32.864	10.283	2.666	-1.739	-5.867	-27.520
IIe2_18	-29.765	7.510	2.676	-2.296	-5.057	-26.931
IIe2_19	-32.497	8.442	3.048	-1.086	-5.720	-27.812
IIe2_20	-29.615	7.882	2.781	-2.583	-5.084	-26.619

VDW: van der Waals contribution (see Supporting Methods)
ELEC_R: Electrostatic contribution (Receptor)
ELEC_L: Electrostatic contribution (Ligand)
COULOM: Coulombic contribution
APOLAR: Apolar contribution
