# Supplementary figures and images for: Protein-Protein Interaction Antagonists as Novel Inhibitors of Non-Canonical Polyubiquitylation
Source: PLoS One. 2010 Jun 30;5(6):e11403. doi: 10.1371/journal.pone.0011403 (PMC2894972; doi:10.1371/journal.pone.0011403)

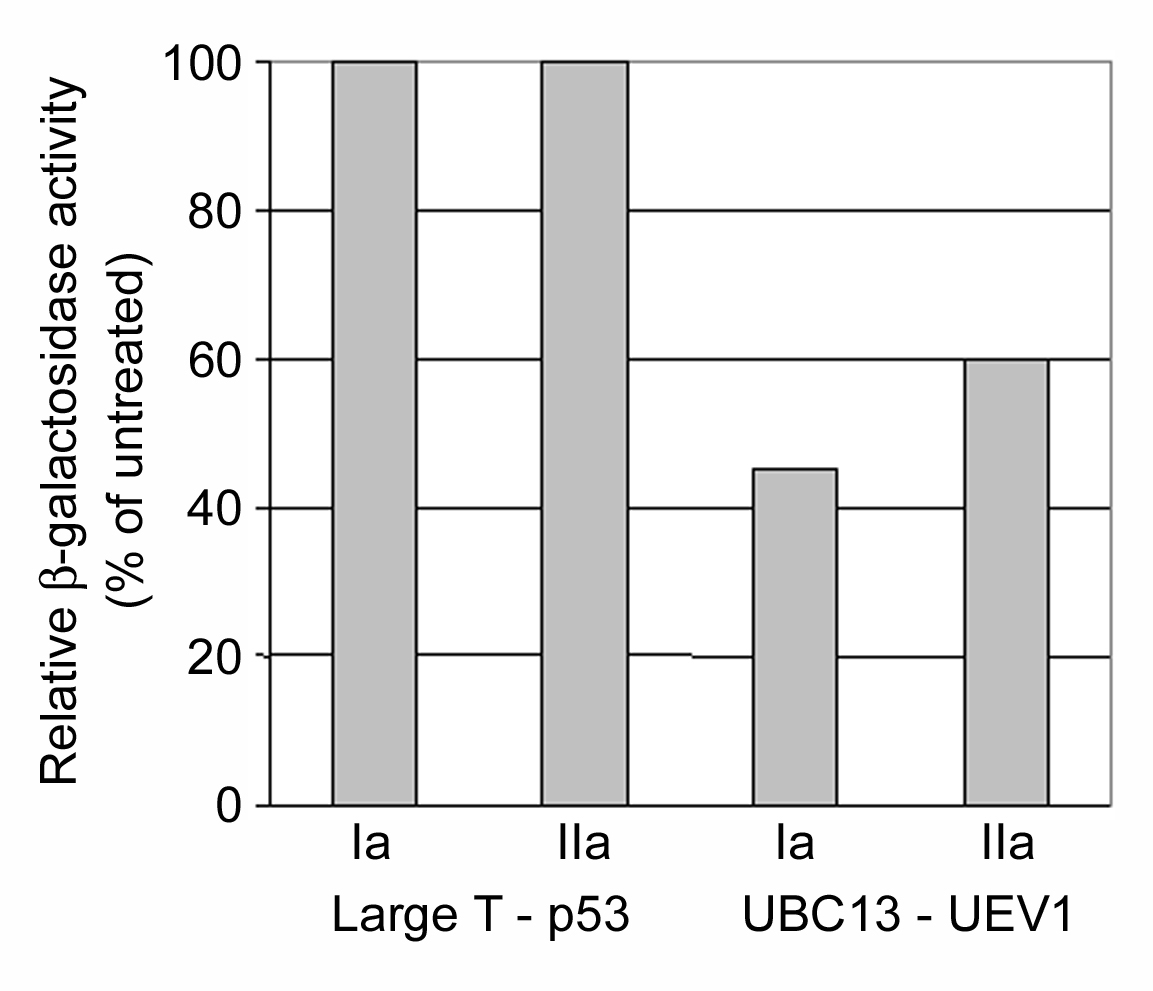

Supplement: Figure S1 — Antagonism by compounds Ia and IIa of the Ubc13-Uev1 interaction, assayed by yeast two-hybrid. Yeast cells (AH109 strain), co-transfected with pBD-Ubc13 and pACT2-Uev1 and grown in quadruple selection medium, were incubated overnight with 100 µM of cyclic compounds Ia or IIa, and assayed for α-galactosidase activity as a semiquantitative measure of strength of interaction. Values were normalized against those of cells incubated with a control, unrelated cyclic compound. As an additional control, yeast cells harboring large T and p53 were assayed in parallel under identical treatments and conditions. (1.17 MB TIF) [file pone.0011403.s004.tif]

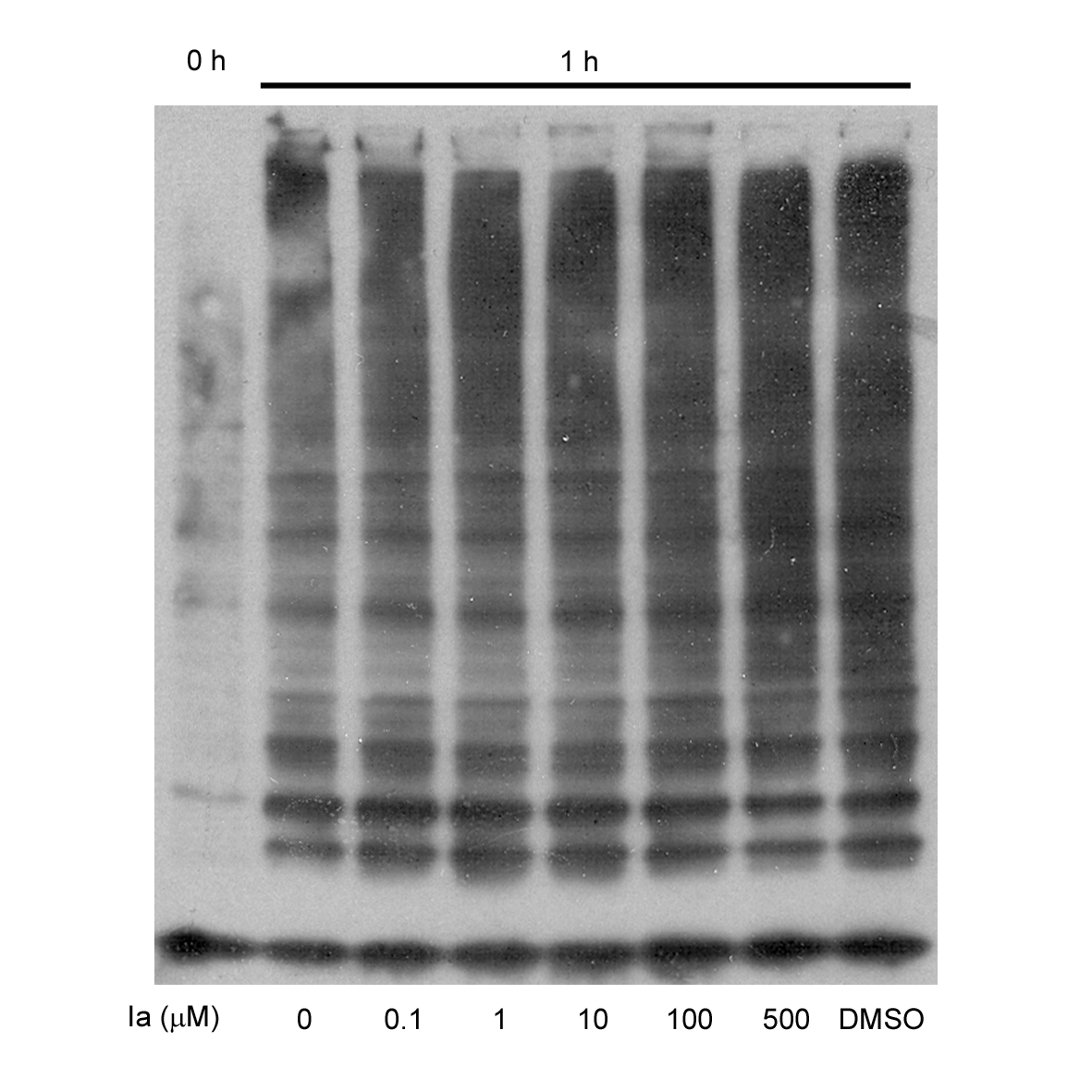

Supplement: Figure S3 — Compound Ia does not inhibit Ubc4-dependent polyubiquitylation of proteasome-associated components. Components of the proteasome holoenzyme undergo K48-based polyubiquitylation in the presence of the ubiquitin ligase Hul5 and the E2 enzyme Ubc4 in a 1-h reaction. Compound Ia does not inhibit the formation of Ubc4-dependent high molecular weight ubiquitin adducts at any of the concentrations tested. DMSO denotes the addition of the solvent at the concentration equivalent to that added when using the maximum concentration of compound Ia used in this experiment (500 µM). (1.42 MB TIF) [file pone.0011403.s006.tif]

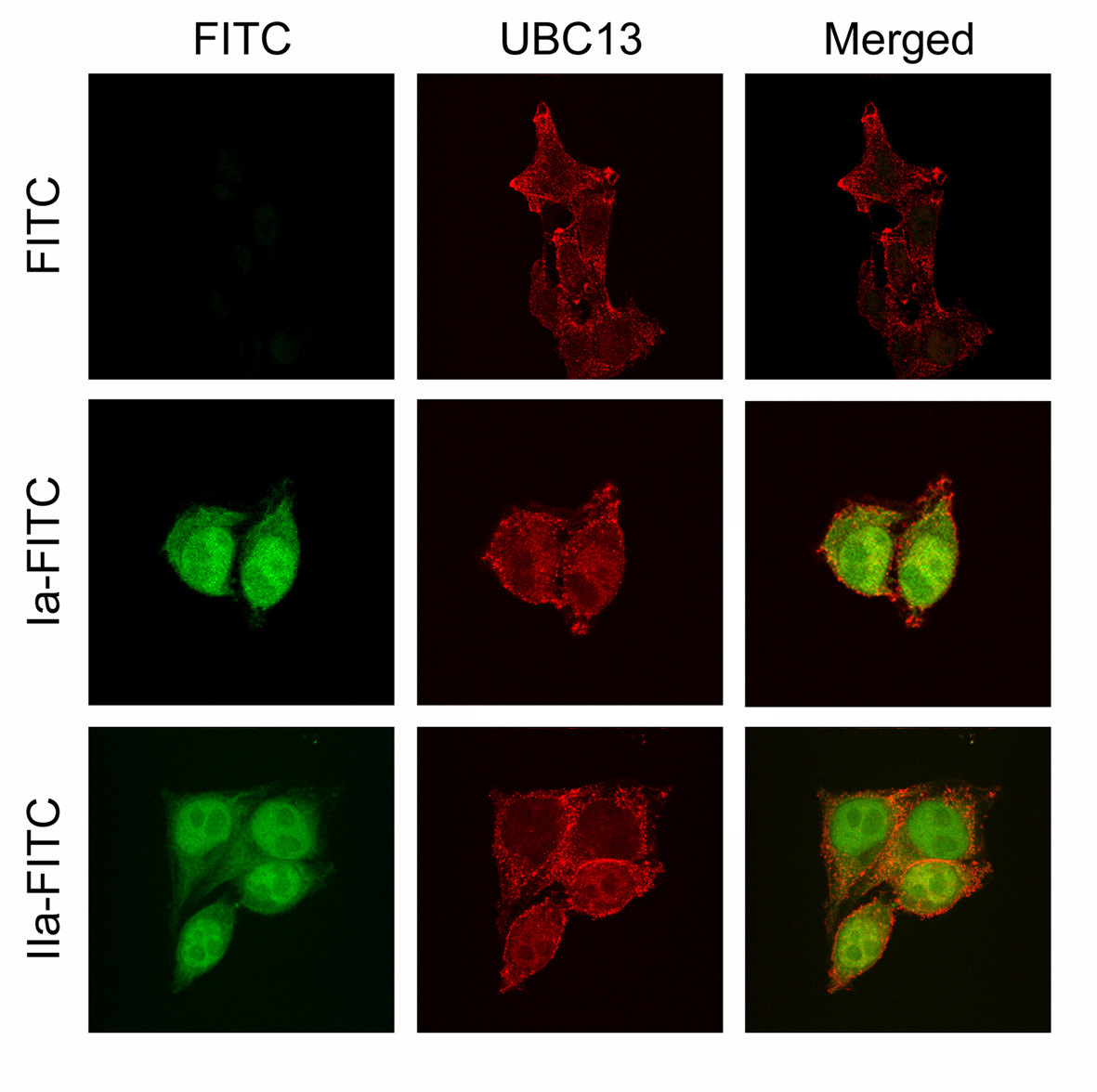

Supplement: Figure S5 — Uptake by mammalian cells of fluoresceinated compounds Ia (Ia-FITC) and IIa (IIa-FITC). HeLa cells, grown on sterile coverslips, were incubated overnight with 100 µM of either Ia-FITC or IIa-FITC, and processed for immunocytochemistry for detection of Ubc13. As a control, HeLa cells were incubated with unconjugated FITC. (4.19 MB TIF) [file pone.0011403.s008.tif]

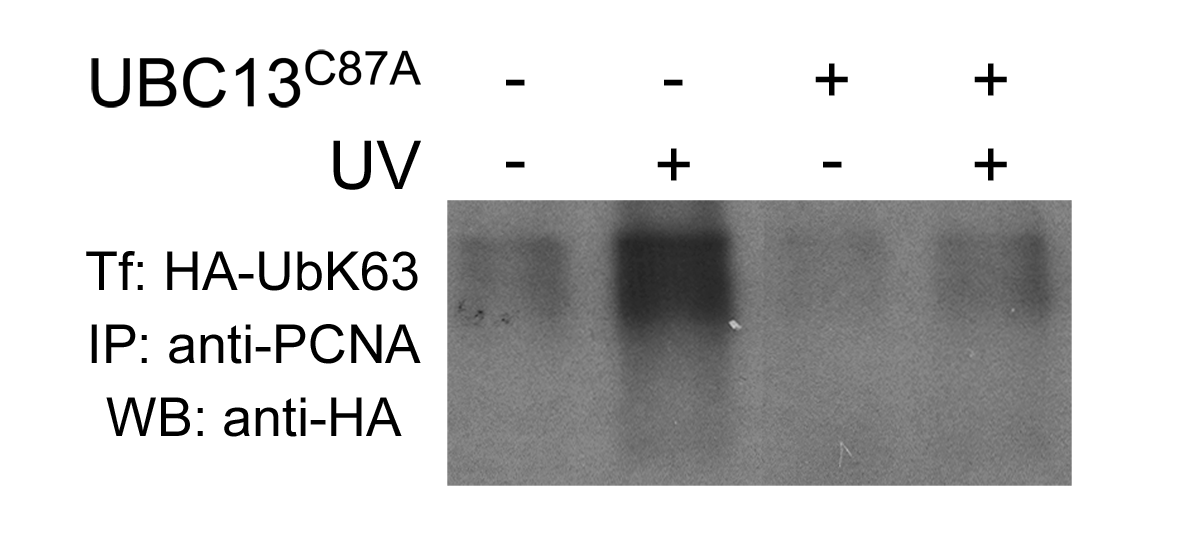

Supplement: Figure S6 — UV-induced, K63-type polyubiquitylation requires enzymatically active Ubc13. PCNA undergoes K63-based polyubiquitylation upon UV irradiation, which is inhibited by transfection of a dominant-negative form of Ubc13 (Ubc13C87A). HeLa cells were transfected with HA-UbK63, together, or not, with pcDNA3.1-Ubc13C87A. After a 24-h preincubation with compound Ia (1 µM), cells were exposed, or not, to UV radiation (60 J/m2), lysed, immunoprecipitated with anti-PCNA, and K63-based polyubuiquitin chains detected by Western blotting with anti-HA. (0.65 MB TIF) [file pone.0011403.s009.tif]

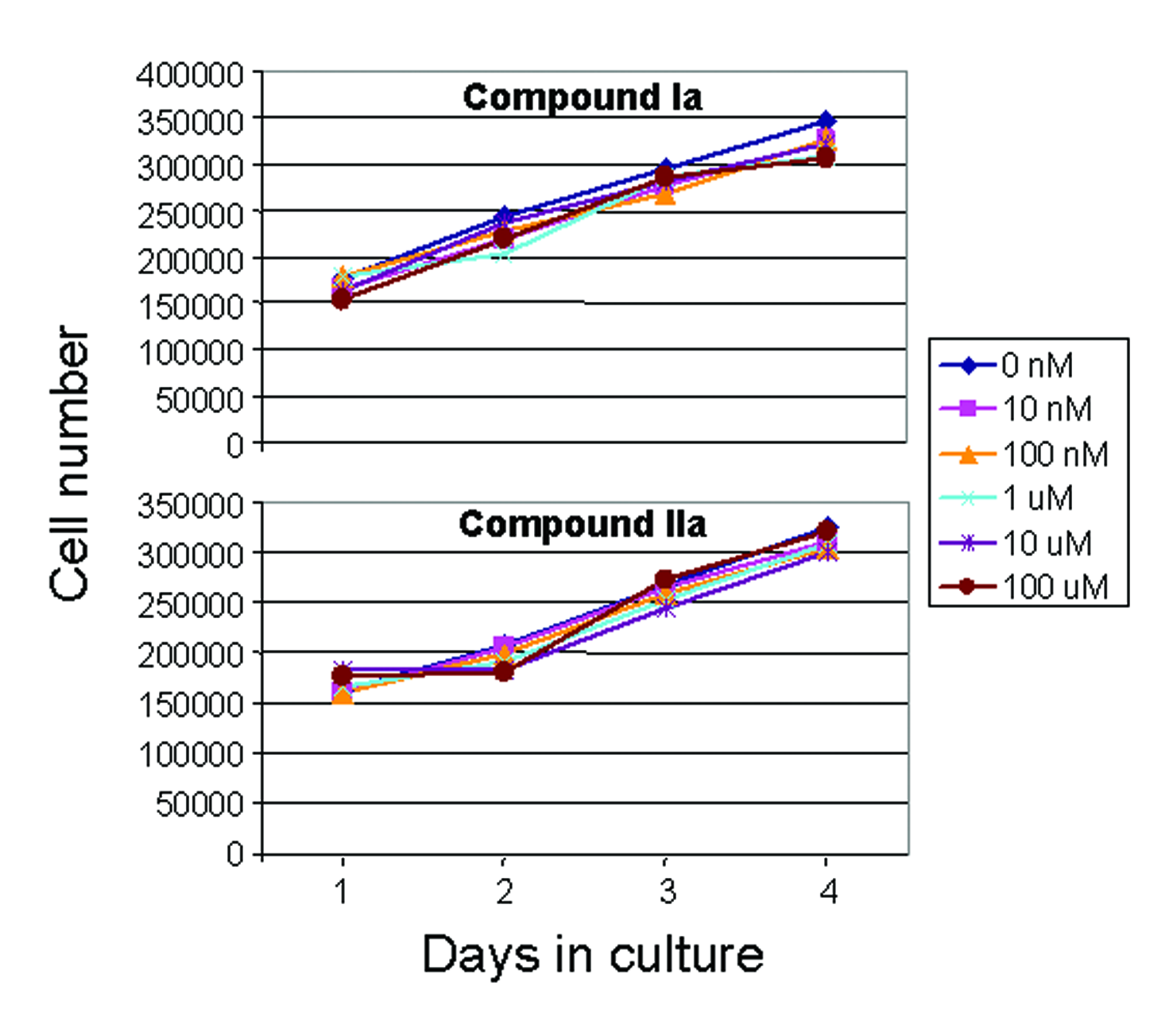

Supplement: Figure S7 — Growth curves of HeLa cells incubated with cyclic compounds Ia (top) or IIa (bottom). Cells were grown for up to 4 days in the presence of varying concentrations of either cyclic compound, freshly added every 48 h, and cell numbers determined by the CyQuant procedure. Shown are average values for each time point and treatment condition, which were done in octuplicate. (7.72 MB TIF) [file pone.0011403.s010.tif]
